# Physical and electrophysiological motor unit characteristics are revealed with simultaneous high-density electromyography and ultrafast ultrasound imaging

**DOI:** 10.1038/s41598-022-12999-4

**Published:** 2022-05-25

**Authors:** Marco Carbonaro, Kristen M. Meiburger, Silvia Seoni, Emma F. Hodson-Tole, Taian Vieira, Alberto Botter

**Affiliations:** 1 Laboratory for Engineering of the Neuromuscular System (LISiN), Department of Electronics and Telecommunication, Politecnico di Torino, 10129 Turin, Italy; 2 PoliToBIOMed Laboratory, Politecnico di Torino, 10129 Turin, Italy; 3 Biolab, Department of Electronics and Telecommunications, Politecnico di Torino, 10129 Turin, Italy; 4 Musculoskeletal Sciences and Sports Medicine Research Centre, Department of Life Sciences, Manchester Metropolitan University, Manchester, M15 6BH UK

**Keywords:** Biomedical engineering, Neurophysiology

## Abstract

Electromyography and ultrasonography provide complementary information about electrophysiological and physical (i.e. anatomical and mechanical) muscle properties. In this study, we propose a method to assess the electrical and physical properties of single motor units (MUs) by combining High-Density surface Electromyography (HDsEMG) and ultrafast ultrasonography (US). Individual MU firings extracted from HDsEMG were used to identify the corresponding region of muscle tissue displacement in US videos. The time evolution of the tissue velocity in the identified region was regarded as the MU tissue displacement velocity. The method was tested in simulated conditions and applied to experimental signals to study the local association between the amplitude distribution of single MU action potentials and the identified displacement area. We were able to identify the location of simulated MUs in the muscle cross-section within a 2 mm error and to reconstruct the simulated MU displacement velocity (cc > 0.85). Multiple regression analysis of 180 experimental MUs detected during isometric contractions of the biceps brachii revealed a significant association between the identified location of MU displacement areas and the centroid of the EMG amplitude distribution. The proposed approach has the potential to enable non-invasive assessment of the electrical, anatomical, and mechanical properties of single MUs in voluntary contractions.

## Introduction

Muscle contraction is associated with a cascade of electrochemical and mechanical events, from the excitation of a motor unit’s (MU) muscle fibers to the binding of actin-myosin and thus generation of muscle force. During both isometric and anisometric contractions in-vivo, the tensile force generated is accompanied by displacement of the contracting and non-contracting fibers^1^. Different methods have been used to assess both muscle excitation and the associated tissue displacement. Electromyography (EMG), for instance, is the gold standard for assessing muscle excitation and neural drive with either intramuscular or surface electrodes^2,3^. Ultrasound (US) imaging, conversely, is often applied to study muscle properties both from anatomic (e.g. tissue architecture and texture) and functional (e.g. muscle contraction patterns, tissue elasticity and muscle anisotropy) perspectives^4–7^. Individually both techniques provide a partial description of the complex electromechanical phenomena leading to muscle force production. When combined, therefore, EMG and US techniques have the potential to provide a more detailed and complete description of the events underpinning the generation of muscle force and more generally, into our understanding of the muscle function^8–11^.

Recently, owing to technological developments of both techniques, the value of a combined EMG-US approach has been extended to the motor unit (MU) level. Over the past two decades, advancements in the detection and processing of surface EMGs from multiple muscle locations (high-density surface EMGs; HDsEMG), have led to improvements in the understanding of how the central nervous system controls the activation of MU populations during force production^12–14^. These results were possible because of the capability of HDsEMG to describe how neural excitation distributes and evolves over the muscle surface. In this regard, evidence on the regional excitation of skeletal muscles has been garnered over the past 20 years both at global^15–18^ and at single MU level^19–22^. The often implicit assumption in these studies is that EMGs with high amplitude are associated with excitation of a spatially localised site underlying the electrode array^23^. The presumptive logic associating local changes in EMG amplitude with local changes in muscle excitation has been substantiated in simulated conditions^24,25^ and, more recently, by local EMG responses to muscle percussion^26,27^. The study of the association between the properties of the EMG amplitude distribution and the location of tissue excitation within the muscle requires a method to assess in vivo muscle tissue displacement associated with the activation of a spatially localized group of fibers. Of pivotal importance in this regard is the possibility of sampling US images at high frame rates (ultrafast ultrasound imaging), allowing to identify displacements of muscle tissue taking place within intervals as short as 0.2 ms^28^. Using ultrafast US imaging, Deffiuex and colleagues^29,30^ were able to describe the displacement of localized regions of muscle during electrically-elicited contractions, suggesting the fibers of the stimulated MU population were grouped within a small muscle region. Incipient accounts furthered the potential of ultrafast US imaging to the assessment of muscle movements related to single MU activation in voluntary contractions. Based on a spatio-temporal decomposition approach, for example, Rohlén et al. proposed and validated a procedure for identifying the firing instants of single MUs during low force voluntary contractions from temporal and spatial variations in the tissue velocity in US images^31^, similar to information extracted from decomposition of EMG signals^32^. This technique allows to assess the location within the image, and therefore within the muscle, where fibers of single MUs are most clearly represented^33^. In light of these advancements, and given the different spatial and temporal sensitivity of EMG and US to MU activation^34^, a method that integrates both techniques may help enlighten the notion of regionalized muscle excitation in skeletal muscles and, in a more general view, enable the study of the spatio-temporal association between electrical and mechanical properties of active MUs.

In the present paper we therefore propose an integrated method to study the local association between the representation of action potentials of single MUs in surface EMGs and the corresponding representation in US images. Specifically, we extract single MU firings from HDsEMG^35^ and use this information to identify the corresponding muscle tissue displacements in US images by adapting the approach proposed by Rohlén and colleagues^31,33^. The method is tested in simulated conditions and applied to experimental signals to determine if there is a regional correspondence between Motor Unit Action Potential (MUAP) amplitude and tissue displacement. If excitation of a single motoneuron results in the excitation and thus movement of fibers spatially grouped within the muscle, we would expect to observe a significant association between location in EMG amplitude distribution and location of tissue movement in US images. In addition to substantiating the value of HDsEMG for the study of regional, muscle excitation, our proposed approach enables in-vivo electromechanical characterization of single MUs, thus providing a framework for studies aimed at improving our understanding of how forces are translated from the individual motor units through to the joint torques.

## Methods

The “Methods” section is organized into five sub-sections. In the first sub-section, we provide an overview of the algorithm integrating HDsEMG and ultrafast US herein proposed to study the spatial localization of MUs in the muscle cross-section. Secondly, we describe the simulation approach adopted to systematically assess the algorithm performances. Thirdly, we describe the experimental protocol used to assess the spatial association between mechanical and electrical MU responses, and in the last two sections we describe the data analysis and statistical methods for both simulated and experimental data.

### Algorithm overview

**Figure 1:**
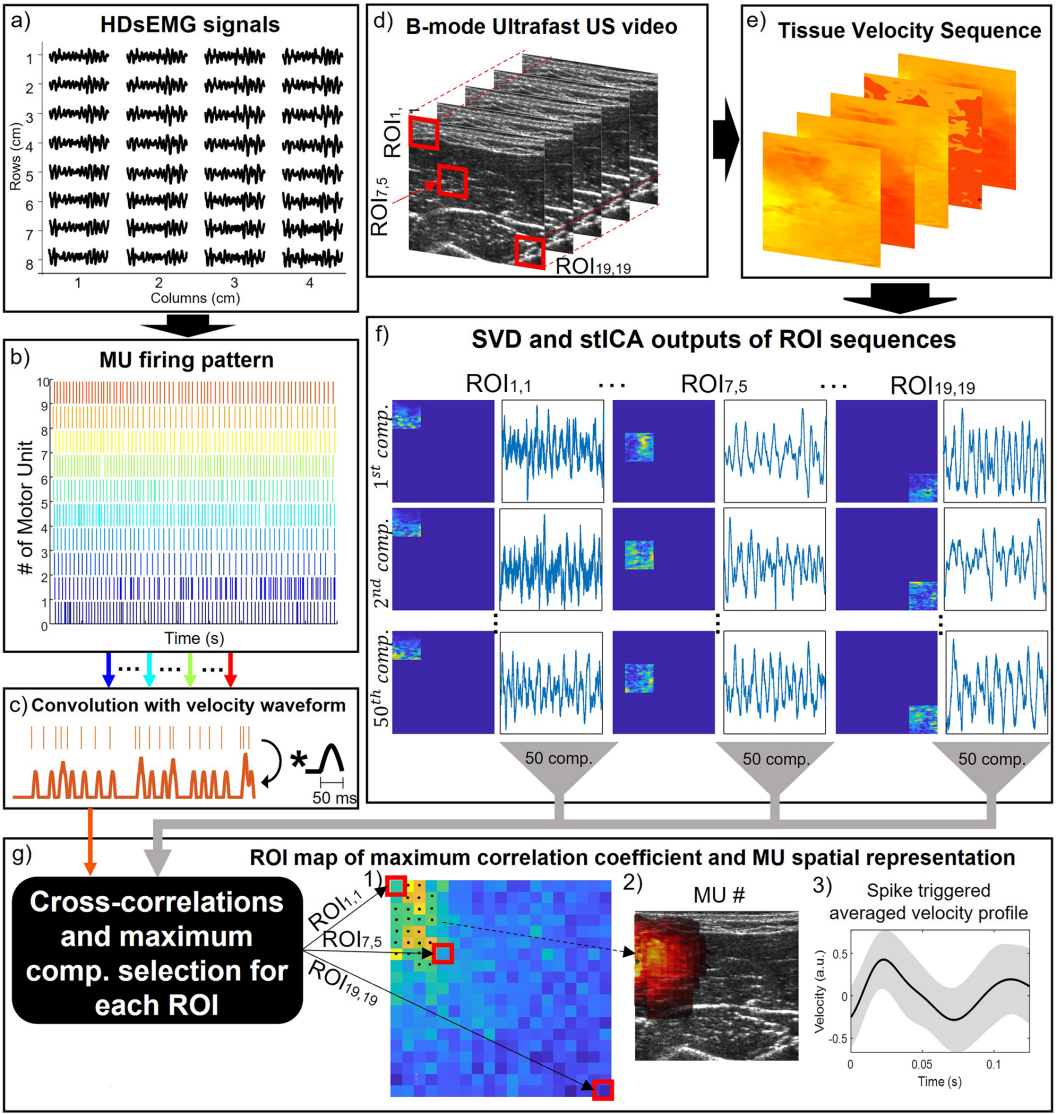
Overview of the proposed algorithm. (**a**) HDsEMG signals of a grid of electrodes. (**b**) Decomposed MU firing pattern from HDsEMG signals. (**c**) Generation of the train of MU displacement velocities through the convolution of the MU firing pattern with the synthetic velocity profile. (**d**) Ultrafast US B-mode sequence with 3 examples of ROI used in the analysis. (**e**) Tissue Velocity Sequence estimated using 2D autocorrelation approach. (**f**) Output of the Singular Value Decomposition and spatio-temporal Independent Component Analysis of the three example ROIs with 50 components (comp.) each composed by a time course (temporal component) and a correspondent image (spatial component). (**g**) Integration between EMG and US variables: cross-correlations between all the temporal components of the tissue velocity sequence of the ROIs and the synthetic train of MU displacement velocities, and example outcome for a single MU. (1) Map of maximum correlation coefficient of all the ROIs (19 × 19). The algorithm extracted the component with the maximum correlation within ± 20 ms time lag for each ROI. The selected ROIs (cluster) are highlighted with black dots. (2) heatmap of the MU spatial representation in US images extracted summing the spatial components of the most correlated ROIs in the coefficient map (black dots); (3) spike triggered averaged velocity profile (black solid line) and its standard deviation (grey band).

**Figure 2:**
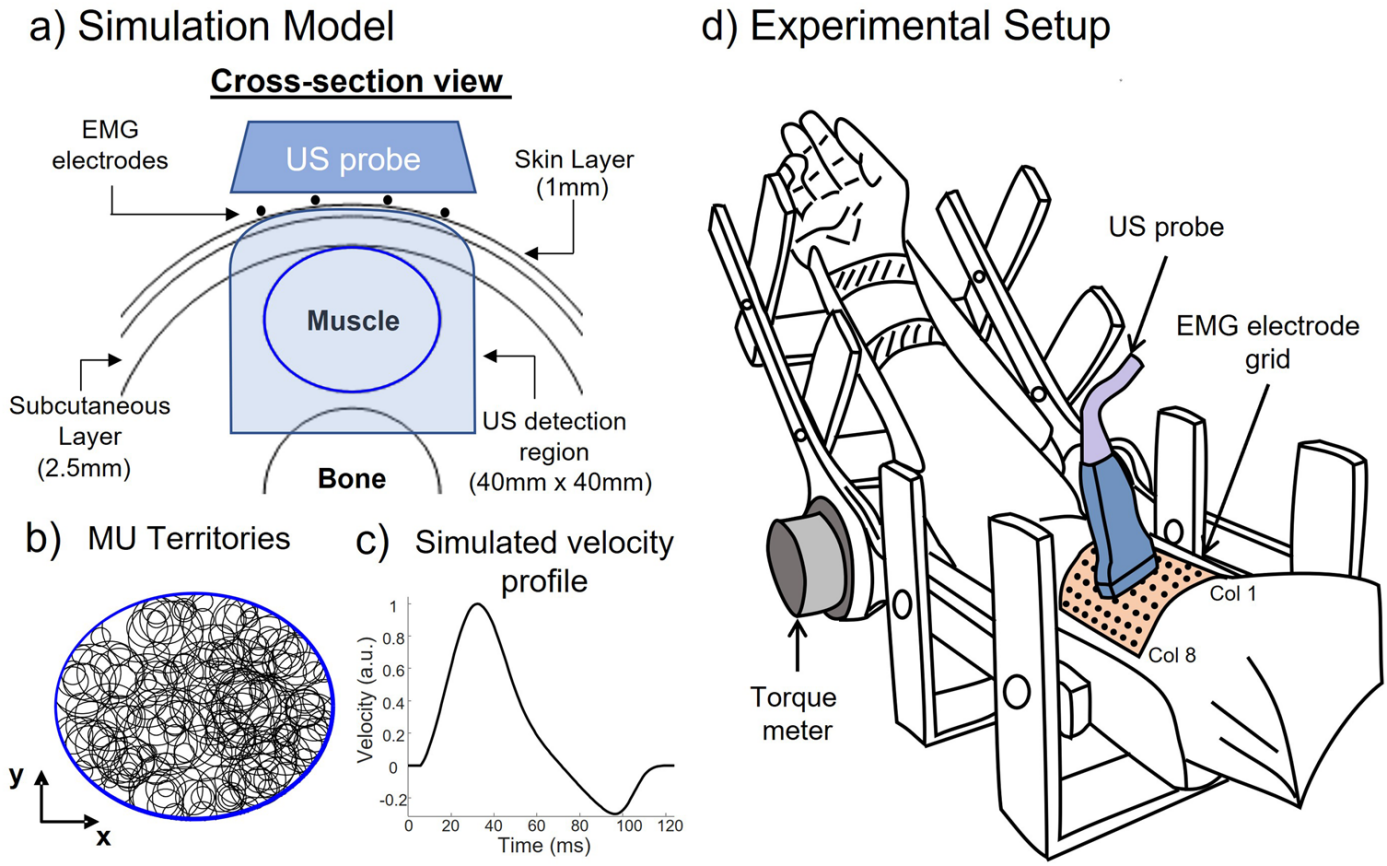
(**a**) Simulation model. Cross-section of the Cylindrical volume conductor model of the limb: 1 mm skin layer, 2.5 mm subcutaneous layer and elliptical-shaped muscle (598 mm^2^ physiological cross-section area). The blue-shaded area is the US detection region (40 mm × 40 mm), including the whole muscle. (**b**) Distribution of MU territories (area range 5–44 mm^2^) inside the simulated muscle. (**c**) Normalized velocity profile of the tissue velocity simulation model. (**d**) Experimental setup overview. The right arm was positioned inside an isometric brace for torque measurements. A grid of 64 EMG electrodes (8 × 8, 1 cm inter-electrode distance) was placed over the biceps brachii. The US probe was positioned between the two central rows of the electrode grid.

The approach for the assessment of the spatial association between individual MUAPs and the corresponding representation in US images is schematically described in Fig. 1. Briefly, the algorithm is based on the combined analysis of HDsEMG (Fig. 1a) and 2D tissue velocity sequences (TVS), computed from ultrafast US images of the muscle cross-section (Fig. 1d,e). From HDsEMG decomposition we obtain the firing instants of individual MUs^35^ (Fig. 1b). For each MU we then compute the convolution between its firing instants and the synthetic waveform representing the average velocity profile of contracting fibers in the superficial-deep axis. This convolution produces what we refer to as the *train of MU displacement velocities* (cf. thick line in Fig. 1c). Afterwards, we apply the spatio-temporal independent component analysis (stICA) to the tissue velocity sequence of each Regions Of Interest (ROI) defined across the whole image (Fig. 1f), thus obtaining the time courses and the corresponding images of the independent components (hereafter referred to as *temporal and spatial components* respectively). For each ROI, we identify the temporal component showing the greatest cross-correlation with the train of MU displacement velocity. Finally, the corresponding spatial components, are used to identify the spatial representation in US images associated with each decomposed MU (Fig. 1g). In the following paragraphs we provide the details concerning the TVS computation and analysis, and its integration with the output of HDsEMG signal decomposition.

#### Tissue velocity sequence computation and spatio-temporal decomposition

Previous reports in literature showed that the analysis of the TVS of muscle cross section allows quantification of the deep-superficial movements of bundles of contracting fibers^30,31,33^. Here we computed the TVS along the US beam axis from radio frequency (RF) signals through autocorrelation in the time (pulse transmissions) and space (depth samples) dimensions^36^. Based on^31^ we used a 1 mm maximal displacement between subsequent images and a sliding window of 10 ms. After velocity values were obtained for each pixel in the image (space) and for each frame (time), velocity profiles in space and time were filtered. A spatial 2-D median filter (1 × 1 mm kernel) was applied to each frame, attenuating the contribution of isolated pixels with spuriously high velocity values. Then, the temporal evolution of each pixel was high pass filtered at 5 Hz (4th order Butterworth filter) to attenuate slow movements not associated with muscle contraction^31^. Afterwards, the smoothed TVS was processed by sliding a ROI^33^ of 12 × 12 mm over the full image (40 × 40 mm) (Fig. 1d). The ROI size and sliding step were selected based on the expected size of MU territories in biceps brachii, and on the spatial resolution of the correlation maps obtained from the sliding ROIs. Specifically, the ROI size is assumed to contain portions of all fibers of a single, contracting MU, whose maximal territory should amount to roughly 80 mm^2^ (territory diameter < 10 mm^33^), and thus should minimize the contribution of other sources to the velocity values. The sliding step was 1.6 mm in both directions (2.56 mm^2^), which was expected to provide roughly 30 samples (i.e. ROIs) per image within the territory of the largest MU in the skeletal muscle investigated in the experimental protocol (biceps brachii MU: 80 mm^2^). We applied singular value decomposition (SVD) to every ROI and retained the 50 most significant eigenimages and corresponding eigensequences. Finally, spatio-temporal independence between the sets of eigenimages and eigensequences was optimized with independent component analysis (ICA)^37^ obtaining 50 spatial components (i.e. images) and corresponding temporal components (i.e. time courses) per ROIs (Fig. 1f). The number of components was higher than that used in previous studies^33^ to increase the likelihood of isolating unique contributions of single MUs to the tissue velocity sequence.

#### Assessing the spatial representation of single MUs in US images

We obtained the synthetic trains of displacement velocities by convoluting the firing patterns of individual MUs with a synthetic waveform, corresponding to the velocity profile of deep-superficial movements of contracting fibers in the US image (Fig. 1c). We specifically considered the positive half of a sine wave, reproducing how fast MU fibers move in the deep-superficial direction in response to a single, end-plate depolarization event. The duration of the synthetic wave was 50 ms for all MUs according to the experimental velocity profile found by Deffieux and colleagues^30^. Afterwards, we computed the cross-correlation between these trains and each of the 50 temporal independent components obtained for each ROI. For each ROI we selected the component with the maximum correlation within a ± 20 ms time lag^38^. This procedure provided a map of correlation coefficients for each decomposed MU (Fig. 1g), in which each pixel lays within a ROI and was given a color scaled with the peak of the cross-correlation. We segmented the correlation map to retain ROIs with correlation values higher than 50% and then identified the largest group (cluster) of connected ROIs with the greatest mean correlation value. The independent spatial components of the ROIs corresponding to the identified cluster were summed to obtain the spatial representation of single MUs in US images (also referred to as *MU displacement area*). The threshold used to identify the cluster of connected ROIs was selected based on preliminary analysis in simulated conditions (see “Simulations” subsection) indicating that 50% was the value leading to the smallest error between the centroids of the simulated MU territory and the estimated MU spatial representation. The temporal components of the identified cluster were averaged to obtain the mean time evolution of the contracting tissue of a certain MU. The tissue displacement velocity profile, hereafter referred to as *velocity profile*, was computed from the time evolution of the contracting tissue through spike triggered averaging based on the MU firing pattern. Each MU was therefore characterized by its spatial representation in the US images and its velocity profile.

### Simulations

The algorithm described in the previous sections was initially tested in simulated conditions to demonstrate a proof of concept. We simulated the excitation of a population of MUs during low-level voluntary contractions and corresponding cross-sectional tissue velocity image sequences. The position of the simulated MU territories was compared with the position of the area identified in the image sequences using the proposed algorithm.

The simulations were based on a cylindrical volume conductor model with skin-parallel fibers^39^ implemented in Matlab software (R2020b, The MathWorks Inc., MA, USA). The cylindrical volume conductor consisted of an anisotropic muscle layer, an isotropic subcutaneous tissue, a skin layer and a muscle with elliptical, cross-sectional area (CSA) (Fig. 2a). The parameters of the anatomical model are reported in Table 1 and were set to represent human biceps brachii (BB) muscle^40^. The territories of MUs were modelled as circular and distributed randomly throughout the muscle (Fig. 2b). Afterwards, a model of recruitment of a population of MUs^41^ was applied to simulate a constant percentage of maximum voluntary contraction (MVC). Specifically, we simulated five steady contractions of ten seconds, each including a progressively greater number of MUs and thus a greater MVC percentage^41^ (Table 2).

**Table 1:** Parameters of the cylindrical model.

	Model parameter	Value
Motor unit properties	Fiber density	~ 135 fib/mm^2^
	Discharge rate	8–15 pps
	CoV of interspike interval	15%
Muscle properties	Number of motor units	200
	Number of muscle fibers	80,000
	Number of MU fibers (range)	150–1500
	Muscle CSA	598 mm^2^
Limb properties	Skin thickness	1 mm
	Subcutaneous tissue thickness	2.5 mm
	Bone (radius)	20 mm

**Table 2:** Simulated contraction levels.

Contraction #	Number of active MUs	%MVC
1	32	2
2	50	3
3	74	5
4	106	10
5	138	20

The tissue velocity image sequences for the five contractions listed in Table 2 were then simulated by combining the anatomical characteristics of the muscle model (Table 1), the positions of the MU territories, the firing pattern of the simulated contractions, and a model of the mechanical responses of single MUs. This latter model describes the spatio-temporal velocity profiles of the MU fibers in the muscle cross-section in response to a single firing. Briefly, for each MU we considered the simulated velocity profile depicted in Fig. 2c, representing the contraction (positive) and relaxation (negative) phases of a group of excited fibers^30^. We associated this velocity profile to each pixel included in the MU territory and scaled its amplitude with the area of the territory (i.e. the number of fibers, with the fiber density constant across the muscle) using a quadratic law^30^. To simulate the passive transmission of velocity to the surrounding non-active fibers, we used a bidimensional exponential decay centered on the MU territory to scale the velocity profile of nearby pixels that were outside the MU territory. We arbitrarily defined the decay rate so that the tissue velocity at a distance of twice the MU territory radius, is about 37% the velocity at the center of the territory. This procedure provided the spatio-temporal mechanical response of each simulated MU. The interferential tissue velocity sequence of the simulated contractions (Table 2) was obtained by summing all the mechanical responses of single MUs according to the MU firing pattern used for EMG simulations. The frame rate of the simulated image sequences was 1024 fps (frames per second) and the size of the images was 128 × 128 pixels, corresponding to a spatial resolution of 0.3 mm for a field of view of 40 × 40 mm.

### Experimental protocol

To demonstrate the proposed algorithm in real data, ten participants (mean ± SD, 29.2 ± 4.6 years, body mass index 23.0 ± 3.0 m/kg^2^, 6 male, 4 female) with no history of neurological or musculoskeletal impairments or disease were enrolled in the protocol. The study was conducted in accordance with the Declaration of Helsinki and the procedure approved by the Regional Ethics Committee (Commissione di Vigilanza, Servizio Sanitario Nazionale-Regione Piemonte, Torino, Italy). Informed consent was obtained from all participants after receiving detailed explanation of the study procedures.

#### Procedure

The participant was seated on a chair with the right arm fixed within an isometric brace for elbow-joint torque measurement, with the elbow flexed at 135° (180° corresponding to full elbow extension; Fig. 2d).

At the beginning of each experiment, the participant performed three MVCs, while visual feedback of the torque level and verbal encouragement were provided. The maximal torque expressed during the three contractions was selected as the reference value for the MVC. After the application of HDsEMG electrodes and the positioning of the US probe over the muscle belly of BB, the participant was requested to perform, in random order, three 60-s long isometric elbow flexions at 2%, 5% and 10% of MVC. HDsEMG signals were acquired during the entire contraction, while US images were detected for about 9 s in the middle of the acquisition. A rectangular pulse provided by an external generator (StimTrig; LISiN, Politecnico di Torino, Italy) was used to start the US acquisition and was concomitantly acquired by the HDsEMG system to synchronize EMG-US acquisitions^42^.

#### HDsEMG recordings

Muscle activity was recorded with a grid of electrodes designed to allow simultaneous acquisition of EMG and US from the same muscle region (adapted from Botter et al.^43^). The grid of 64 electrodes (8 rows by 8 columns, 10 mm inter-electrode distance) was secured over the BB muscle belly with the columns aligned with the longitudinal axis of the arm, after appropriate skin preparation^44^. The first four columns of the grid were placed on BB short head and the other four on the long head (Fig. 2d). The separation between the two BB heads was identified with US imaging prior to the electrode positioning as described in Pinto et al.^45^. Monopolar EMG signals were detected and conditioned (Bandwidth 10–500 Hz, Gain 46 dB), and sampled at 2048 Hz with 16-bit resolution through a wireless HDsEMG acquisition system (MEACS, LISiN, Politecnico di Torino, Turin, Italy)^46,47^.

#### Ultrasound acquisition

We acquired RF data with a Verasonics Vantage 128 (Verasonics, Inc., Kirkland, WA) US platform, which is a programmable US research platform, together with a Verasonics L11-5v linear array transducer (7.8125 MHz center frequency). A probe holder was fixed on the isometric brace to firmly keep the US probe over the BB muscle, perpendicular to the longitudinal axis of the arm. The probe was placed between the fourth and the fifth row of electrodes and centered with respect to the columns, thus scanning the two heads of the BB in the medio-lateral direction (cross-sectional) (Fig. 2d). A custom MATLAB (MathWorks, Natick, MA) code was used to control the RF acquisition through an external starting trigger and to acquire plane wave data with a frame rate of 2500 fps. The RF data was sampled at four times the transducer center frequency (31.25 MHz) and was reconstructed in post-processing using the standard delay-and-sum (DAS) beamforming method. After computing tissue velocity sequences (see “Tissue velocity sequence computation and spatio-temporal decomposition”), data were downsampled in the axial direction (125 × 125 pixels) such that a final spatial resolution of about 0.3 × 0.3 mm was achieved (field of view of 40 × 40 mm).

### Data analysis

The individual MU firings and the tissue velocity sequence of the muscle excitation during both simulated and in vivo contractions were used as input to the algorithm as described in the “Algorithm overview” sub-section and in Fig. 1.

For the simulations listed in Table 2, the agreement between the output variables of our algorithm and the simulated MU characteristics was quantified as follows: the Euclidean distances between the centroids of the identified MU displacement area and the center of the simulated MU territories were computed to measure the error in the localization of the MU in the muscle cross-section. The zero-lag cross correlation coefficients were used to assess the similarity between the identified velocity profiles and the simulated ones (Fig. 2c).

For the in vivo data, HDsEMG signals were bandpass filtered in the 20–400 Hz band and, when required, residual power line interference were removed^48^. Afterwards, signals were decomposed into individual MU firing patterns with a validated method based on convolution kernel compensation (CKC)^35,49^. The firing pattern of the decomposed MUs was resampled at the sampling frequency of the US images (2500 Hz).

For both simulated and in vivo data we compared the centroids of the MUAP amplitude distributions^25^ with the centroids of the corresponding MU spatial representations in US images, provided by the algorithm.

### Statistics

Statistical differences over the contraction levels in simulated conditions were assessed with one-way ANOVA for both the Euclidean distances and velocity profiles correlation coefficients (significance with p < 0.05).

A simple linear regression of the medio-lateral coordinates of the EMG and identified area centroids of simulated MU was computed to demonstrate the relationship between location in EMG amplitude distribution and location of tissue movement in US images.

A multiple regression analysis (${Y}_{fit}= {\upbeta }_{1}+ {\upbeta }_{2} {\mathrm{x}}_{1} + {\upbeta }_{3} {\mathrm{x}}_{2} + {\upbeta }_{4} {\mathrm{x}}_{1} {\mathrm{x}}_{2}$) on experimental data was implemented to assess the relationship of the medio-lateral coordinate of the US centroids (dependent variable; $Y$) with the medio-lateral position of the EMG centroids and the depth of the identified centroids in the US image (independent variables; ${\mathrm{x}}_{1}$ and ${\mathrm{x}}_{2}$, respectively). We also added an interaction term to check whether there is a significant effect of the depth of the identification with the medio-lateral EMG-US relation. We assumed no collinearity between the independent variables because they are derived from different measurement modalities. Statistical significances were assessed based on 95% confidence intervals of the model coefficients.

All the statistical tests were performed in Matlab (R2020b, The MathWorks Inc., MA, USA).

## Results

### Algorithm performance on simulated data

**Figure 3:**
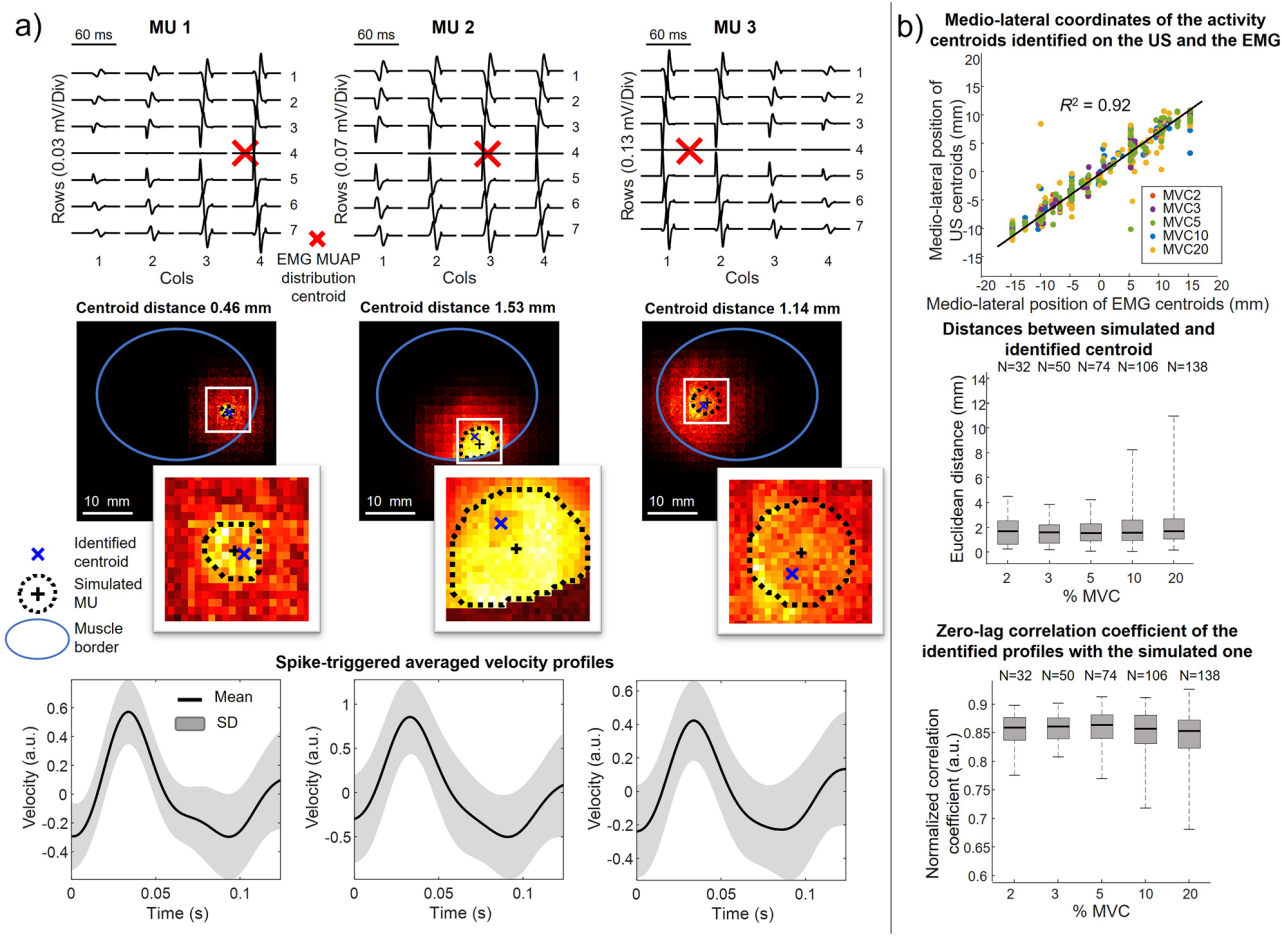
Simulation results. (**a**) Three representative outputs of the algorithm on simulated MUs. From top to bottom: longitudinal differential MUAP decomposed from EMG with the correspondent centroid (red cross); MU spatial representation in US images identified by the algorithm; a zoom of the simulated MU territory (MU center ‘+’ and contour in black dotted line) and the centroid of identified region (blue cross); mean (black solid line) and the standard deviation (grey band) of the spike-triggered averaged velocity profiles related to the correspondent identified region of tissue displacement. (**b**) Group results of all 400 simulated MUs. From top to bottom: scatterplot of the relation between the medio-lateral coordinates of the MUAP centroids (red cross) and the US centroids (blue cross); boxplots of the Euclidean distances (i.e. error) between the center of the simulated MU and the identified centroid over different simulated contractions; boxplots of the zero-lag correlation coefficient of the identified profile with the simulated velocity profile over different simulated contractions.

Figure 3a shows the results of the simulation and the processing of three representative MUs. The figure depicts the single differential MUAPs computed longitudinally (i.e. along the fibers’ direction) (top panel), the corresponding spatial representation on the simulated TVS images (middle panel) and the spike triggered averaged velocity profiles based on the MU firing pattern (bottom panel). The insets in the middle panel are an expanded image, focused on the center (black cross) of the simulated MU territory (dashed black line). The centroid of the identified area falls into the simulated territory and its medio-lateral position is associated with the medio-lateral coordinate of the MUAP centroid (red cross in the upper panel). When considering group data, the medio-lateral coordinates of the MUAP centroid of all MUs (*N* = 400) were highly positively correlated with the medio-lateral coordinates of identified centroids in the simulated image sequences (*R*^2^ = 0.92, p < 0.05). The boxplot shown in Fig. 3b (middle panel) reports the errors (Euclidean distances between the center of simulated MU territories and the centroid of the identified areas) for all the simulated contraction levels. Although the ranges of error distributions increased for the higher contraction levels (0.2–3.8 mm for 3% MVC, 0.1–4.2 mm for 5% MVC, 0.0–8.2 mm for 10% MVC, 0.2–10.9 mm for 20% MVC), no statistically significant effect of the contraction level was observed (*p* = 0.2, *F* = 1.48). The median error across all contraction levels was 1.6 mm (5 pixels). The lower panel of Fig. 3b shows the zero-lag correlation coefficients between the identified velocity profiles and the simulated ones (Fig. 2c) for different contraction levels. The median correlation coefficient across contraction levels was 0.85, indicating strong agreement between the simulated and reconstructed velocity profiles. Similarly to the error results, we did not observe a significant effect of the contraction level (*p* = 0.5, *F* = 0.83) although an increase in the distribution ranges was observed for higher contraction levels.

### Experimental results

All participants completed the protocol and none reported any kind of discomfort or fatigue. A total number of 180 MUs were analysed (mean ± SD, 18 ± 8 MUs per participant, average firing rate: 11.3 ± 2.1 pps), after excluding MUs with less than 20 firing instants in the US acquisition time interval, those with a coefficient of variation of the inter-spike interval larger than 30^50^, and MUs whose action potentials were of dubious nature (c.f. Fig. 4d in Power et al.^51^).

**Figure 4:**
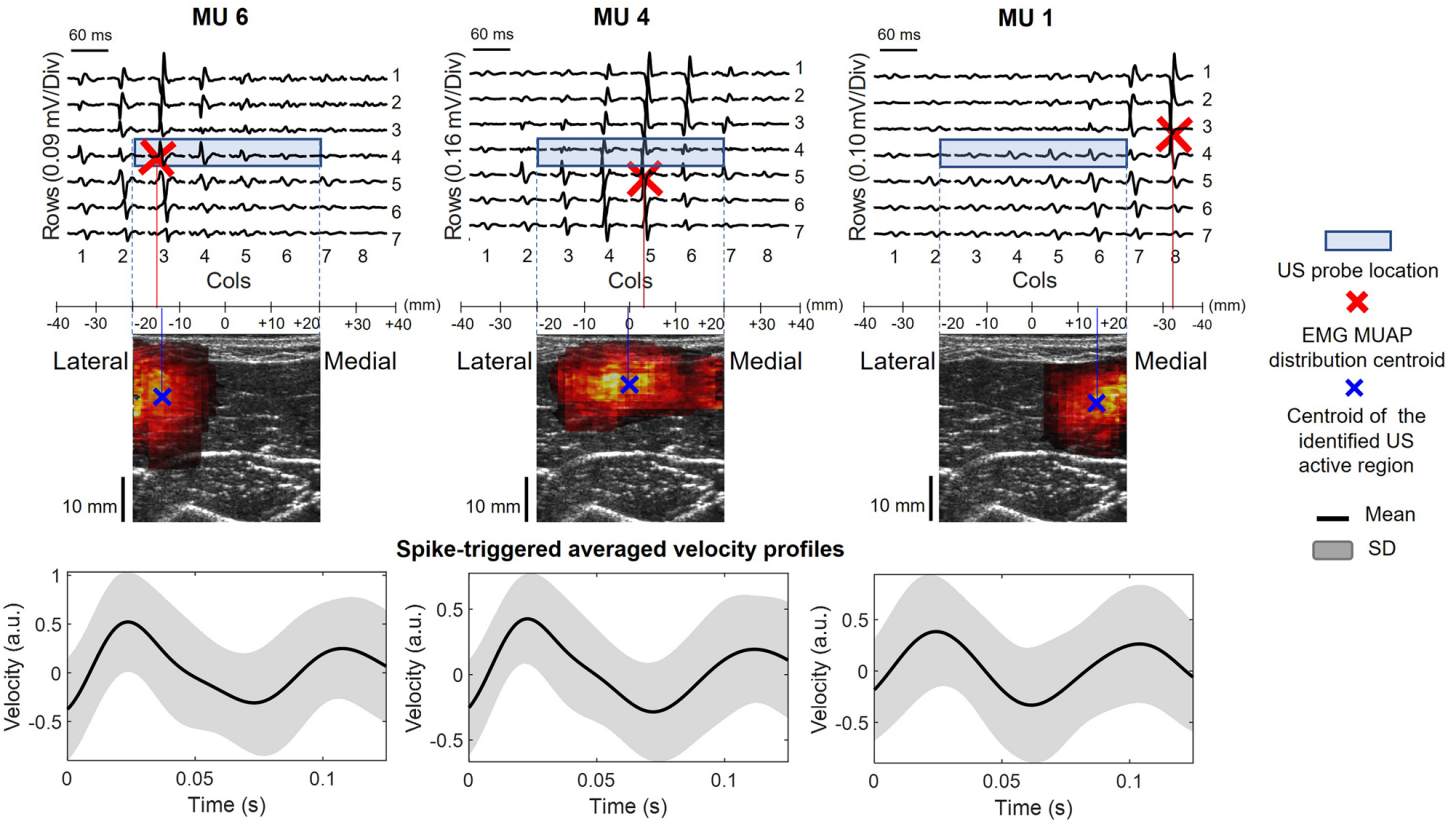
Example of three MUs from biceps brachii during a 2% isometric contraction in one representative subject. From top to bottom: MUAPs decomposed from HDsEMG with the correspondent centroids (red crosses); identified MU spatial representation overlapped on the B-mode images of BB with correspondent centroids (blue crosses); means (black solid line) and the standard deviation (grey band) of the spike-triggered averaged velocity profiles related to the correspondent identified areas.

**Figure 5:**
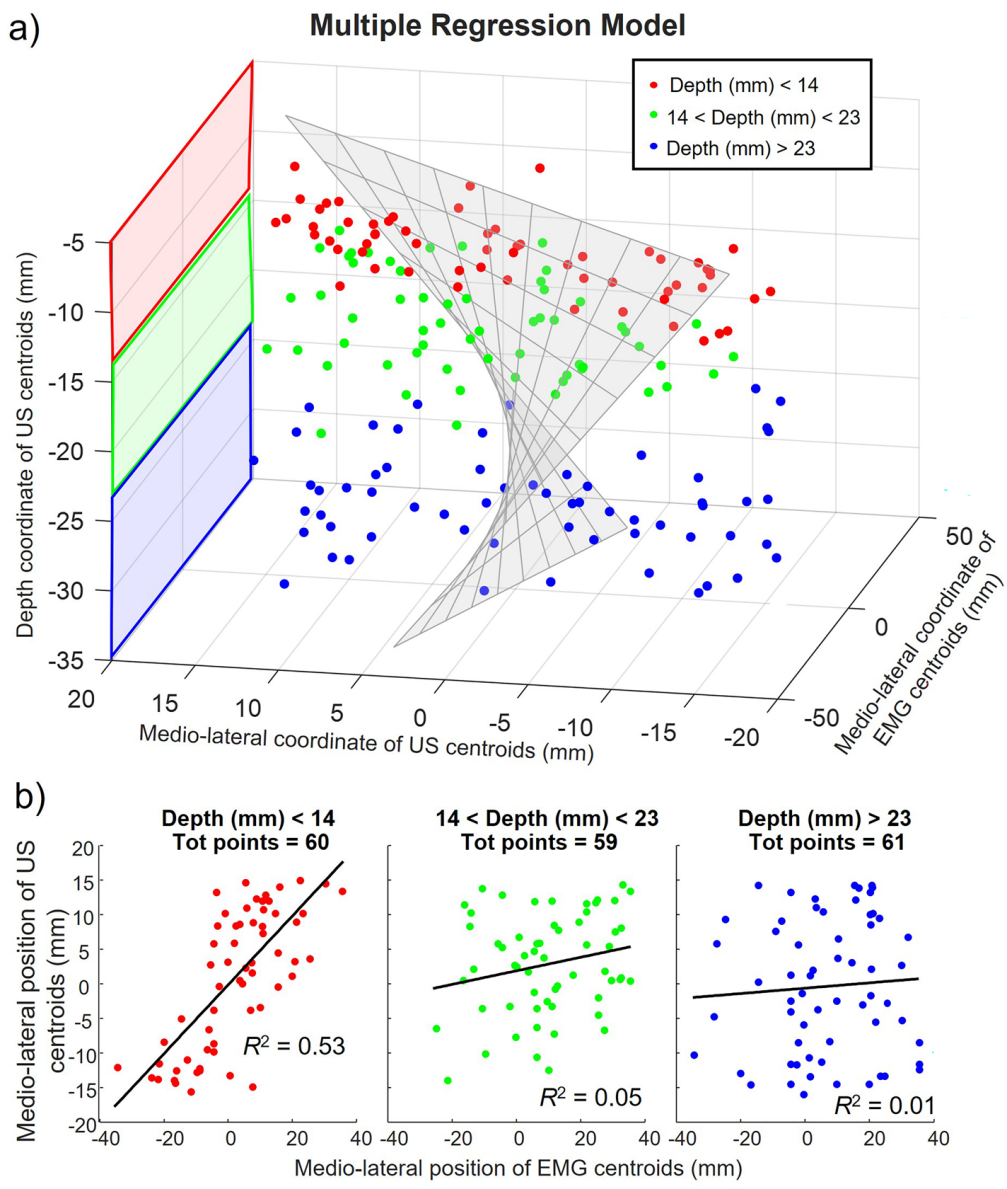
Group results of the experimental data. (**a**) 3D scatterplot of the relationship between the medio-lateral coordinates of centroids in EMG and US identified area, considering the effect of the depth in US. The surface represents the best fit of the multiple regression model. (**b**) Scatterplots of the MU divided by the US identification depth. From left to right: depth < 14 mm (N = 60), depth > 14 mm and < 23 mm (N = 59), depth > 23 mm (N = 61).

Figure 4 shows examples for three MUs decomposed during a 2% MVC contraction in a representative participant. The three MUs were represented differently in the surface EMGs, with MUs #1, #4 and #6 being associated with action potentials distributed respectively medially, centrally and laterally in the electrode array (Fig. 4, top panel). Overall, the centroids of the identified MUAPs were distributed uniformly across the columns (transversal muscle direction), while in the longitudinal direction they were clustered around row 4 (80% between row 3 and 5), where the US probe was positioned. Inspection of US images, with superimposed identified MU areas (i.e. heatmaps), revealed a clear, spatial association between the EMG and US representation of the three MUs. Multiple regression analysis revealed a significant association between the medio-lateral location of centroids in EMG and US (Fig. 5a, R^2^ = 0.20 and *p* < 0.05), regardless of how deep the centroid in US images was located. The confidence interval for the linear coefficient relating medio-lateral coordinate of EMG centroids to US centroids (${\upbeta }_{2}$) ranged from 0.39 to 0.82 (*p* < 0.05). The confidence interval for the coefficient of the depth of the US area relating with its medio-lateral position (${\upbeta }_{3}$) ranged from -0.16 to 0.176 (*p* = 0.94). The confidence interval for the coefficient of depth × EMG centroids (${\upbeta }_{4}$) ranged from 0.01 to 0.03 mm^−1^ (*p* < 0.05). In fact, as depicted in Fig. 5b, the more the US identification is superficial, the more the relationship between the US and EMG identification in the medio-lateral direction is linear. For depths lower than 14 mm the variance explained by the model was 53% (*N* = 60, *p* < 0.05), whereas for depths between 14 and 23 mm it was 5% (*N* = 59, *p* = 0.09) and for depths greater than 23 mm it was 1% (*N* = 61, *p* = 0.6).

## Discussion

In this work, we propose a method to assess the electrical and spatial, physical properties of single motor units in vivo. We have used this method to study the association between the spatial representation of single MUs in surface EMGs and in US images. By integrating HDsEMG decomposition with spatio-temporal decomposition of the ultrasound tissue velocity sequences, we were able to identify the electrical and mechanical responses of 180 MUs during isometric, constant-force contractions. Our results revealed that single MU activation is represented in spatially localised regions of both EMGs and US images. Most importantly, in the medio-lateral direction, we observed a significant association between the skin regions where MU action potentials with greatest amplitude were detected in the electrode array and the muscle regions where the MU mechanical responses were represented in US images. Our results support the notion that HDsEMG is sensitive to local variations in excitation within the muscle, as well as the potential of a combined, EMG-US analysis to study electrical and mechanical properties of single MUs during voluntary contraction.

Our method is based on the identification of the firing instants of single MUs, that are used to obtain the electrical (in EMG signals) and physical (in US images) representation of single MU activation. This method represents an integration between HDsEMG and US signal processing. Indeed, by using HDsEMG and US decomposition separately, it is possible to obtain the electrical^49^ and physical^31^ representation of single MUs. However, this approach would require a validated criterion to associate retrospectively pairs of single MUs identified by the two methods independently. In this regard, the agreement between MU firing instants obtained by two decomposition methods (e.g. needle and HDsEMG) have been previously used^50^, however, in our case, the relatively high number of mismatches between electrical and mechanical firing instants of single MUs^33,34^ seems to limit its current applicability. For this reason, in this study, we rely on the firing instants of single MUs provided by one method as first step of the algorithm (Fig. 1b). Two approaches could have been used to obtain the firing instants of single MUs. Specifically, MUs’ firings could have been identified through decomposition of either HDsEMG^49^ or the tissue velocity sequences obtained from ultrafast US images^31^. We selected the former decomposition method for two reasons. First, although both methods have been validated, validation of HDsEMG decomposition has been assessed more widely and in different experimental conditions^32,49,50^. Second, we expect the decomposition of HDsEMG to have a greater, overall discriminative power than the decomposition of tissue velocity sequences. While both monopolar HDsEMG and ultrafast US imaging have high temporal and spatial resolution, the duration of the physiological events detected by HDsEMG and US imaging differs significantly. In surface EMGs, the duration of single action potentials is often shorter than 30 ms (c.f. Fig. 4) whereas, in US images, the tissue displacements generated in response to a single MU firing last roughly 10 times longer^30^. Owing to the shorter duration of action potentials when compared to the duration of the tissue displacement, superposition of different MUs is more likely to be resolved in surface EMGs than US images. By using the firing instants decomposed from HDsEMG we therefore believe to have assessed the association between the location of electrical and mechanical responses of a broad population of MUs.

Being this the first attempt to assess the MU spatial representation in surface EMG and US images, we first tested the validity of our approach in simulated data. Technical considerations are warranted though before commenting on simulation results. On the one hand, by decomposing monopolar HDsEMGs we indiscriminately assessed both superficial and deep MUs, given that in monopolar derivation the specificity of surface EMGs is remarkably low^52^. On the other hand, we limited our analysis to MUs whose territory was within the detection volume of bipolar EMGs with a 10 mm inter-electrode distance: for circular MU territories with 10 mm radius^33^, the detection volume of these electrodes would include fibers of MUs with territories centred at a maximal depth of 20 mm^53^. Our decision was not only motivated by the fact that 8–10 mm inter electrode distance is often used for detection of EMGs from the biceps brachii muscle (this and other studies^12,14,19,23,53^). Most importantly, our research question presumes that MUs with small territories in relation to the muscle cross-sectional area are represented locally in the surface EMGs. In muscles where fibers run parallel to the skin, like BB (c.f. Fig. 2 in Vieira et al.^23^), this local association is however expected only for superficial MUs. The deeper the MU territory within the muscle the more widely diffused the action potential amplitudes distribute in the transverse direction across the electrode array^54^. Corroborating the view that differential EMGs detected with shorter inter-electrode distances are sensitive to more superficial MUs, our simulation results demonstrated a strong association between the medio-lateral coordinates of centroids of regions where MUs were represented in US images and in the HDsEMGs (Fig. 3). Moreover, our method was able to identify the center of the territory of simulated MUs with a precision greater than 2 mm, regardless of the contraction level and thus of the size of simulated units. Our theoretical approach, based on the use of a validated model for the simulation of EMGs^39^ and on the spatially smoothed simulation of tissue velocity sequences^31^, accounting for movement of both passive and actively contracting fibers, therefore supports the notion that anatomical information on single MUs may be inferred from HDsEMGs for the set of conditions simulated in our model.

Our experimental findings aligned with those from the simulated data. Specifically, the medio-lateral center of the electrode region where the greatest amplitude single MU action potentials were detected was significantly correlated with the transverse coordinate of the associated center of the identified US region (Fig. 5). As anticipated, the strength of this relationship scaled with the depth of the MU’s territory in the US images (c.f. depth coordinate of x in Fig. 4). This likely reflects the diffusion of the potentials, induced by the interposed tissues, leading to the amplitude of deep MUs’ potentials distributing more similarly across the surface EMGs^54^. Therefore deep MUs, with territories centered in different transverse locations, are represented similarly in bipolar, surface EMG, which undermines the discriminative power of this modality. Moreover, owing to a higher sensitivity to superficial sources, surface EMG might be biased towards the contributions from upper portion of deep MU territories, and this may play a role in the observed mismatch between the positions of EMG- and US-based centroids of deep MUs. These possible factors are evidenced by the significant interaction effect between the US image-derived depth coordinate centroids and the medio-lateral coordinate of EMG centroids (Fig. 5a). By decomposing monopolar EMGs, we aimed at identifying the largest possible number of MUs^55^, indiscriminately including superficial and deep units. By grouping units according to their depth representation in the US images we were however able to more clearly evidence the expected association between the transverse, local representation of MUs in US images and in bipolar, surface EMGs. Indeed, the coefficient of determination obtained for superficial MUs was at least twenty times greater than those obtained for MUs where tissue displacements were centered deeper than 14 mm (Fig. 5b).

**Figure 6:**
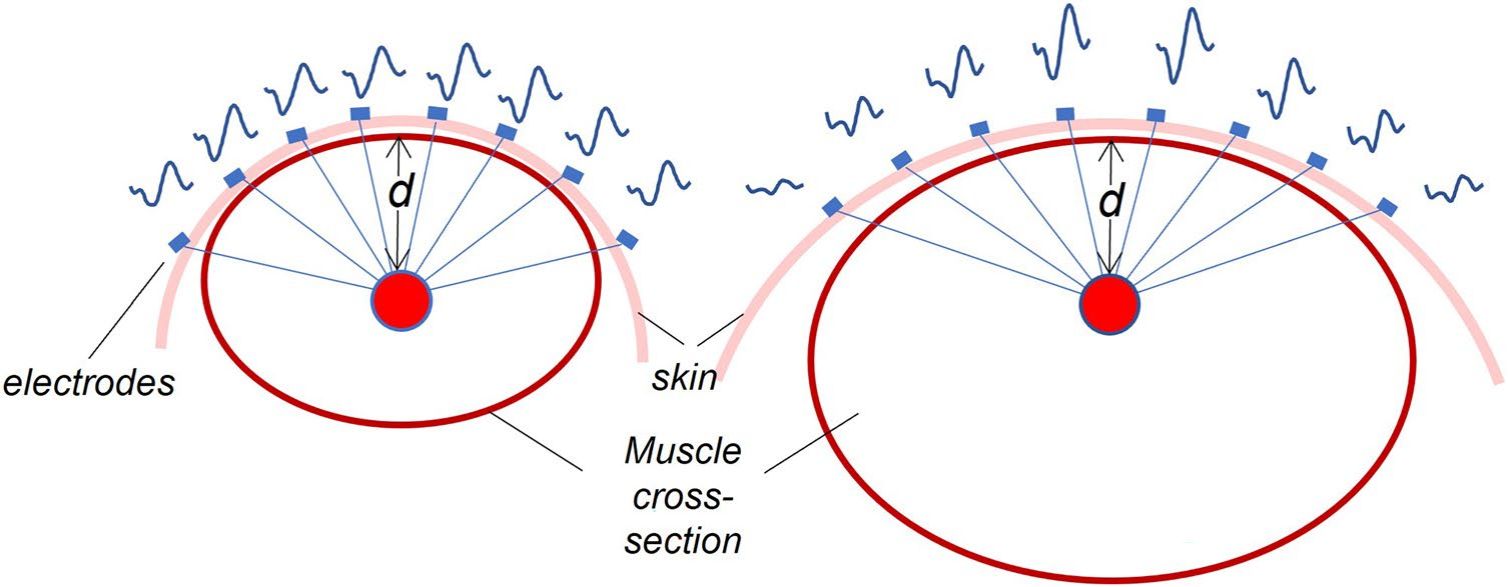
Effect of the muscle physiological cross-sectional area (PCSA) on the transversal MUAP amplitude distribution. Red circles represent the territories of two MUs located at the same depth (d) in two muscles with different PCSA. Right muscle (small PCSA): the distance between active fibers and the skin surface is similar for all the skin locations, leading to a uniform MUAP amplitude distribution across the detecting electrodes. Left muscle (large PCSA): the distance between active fibers and the skin surface changes for different skin locations, leading to a more localized amplitude distribution.

Notwithstanding the stronger spatial association for the more superficial units, experimental results were not as strongly supportive as simulation results. The reasons for this will relate to the effect of additional factors, other than the depth of MUs, on how MU activation is represented in EMG and US. One first key source to mention is inter-individual, anatomical variation. As schematically shown in Fig. 6, for spatially localized MUs centered at equal depths, the distance between electrodes and the center of the MU territory changes according to the size and shape of the surrounding tissue, and so does the transverse representation of action potentials^54^. In Fig. 6, variation is illustrated in terms of different sizes of the muscle cross-sectional area, but it should be noted that any other anatomical source affecting the distance between electrodes and MUs would equally impact on the unit representation in the surface EMG^56,57^. Crucially, this issue affects MUs at different depths, although it is likely more prominent for deeper units. In line with this reasoning, the fixed 10 mm inter-electrode distance used to compute bipolar EMGs may convey a different proportion of fibers of single MUs for different individuals: more superficial units were likely less locally represented in our bipolar EMGs for subjects with a thicker subcutaneous adipose tissue. A second source of variation to consider is our current limited understanding on how well contractions of single MUs can be tracked in US images. Our algorithm found a correspondent mechanical representation in the US for each MU decomposed. However, mismatches between electrical activity and mechanical response have been reported^33,34^, suggesting false positive tissue displacements may have been identified. Currently we are unable to predict and simulate the effect of the major sources of variation in both EMG and US representation of single MUs. Nevertheless, our results document the potential of HDsEMG in revealing spatial differences in muscle excitation and encourage the use of US analysis to assess the anatomical properties of single MUs, with the combination of both techniques having the potential to provide a comprehensive analysis of electromechanical events at the MU level.

The generalization of our results to muscles and conditions other than those tested here may not be straightforward and requires some additional considerations. One first point to consider is that our analysis requires that US images are taken from the muscle CSA, given that we measured the component of the tissue velocity in the depth direction of the US image. Indeed, heatmaps superimposed on the US images in Figs. 3 and 4 are computed from the tissue displacement velocity in the depth direction of the US image and therefore of the biceps brachii CSA. It is expected that these tissue velocities depend on where the contracting fibers are located within the muscle CSA. Whether this interpretation holds for US images taken from planes oblique to the muscle CSA requires further investigation. Of particular interest would be assessing the association between the location of contracting fibers within the muscle CSA and the region with greatest tissue displacement velocity images taken obliquely. A second point of interest is the generalization of the EMG-US association reported here to MUs recruited at greater force levels. Because of the progressive recruitment of a greater number of MUs, and increase of firing rate, both EMGs and tissue velocity sequences become more interferential at higher contraction levels, presumably limiting the ability to discriminate single MUs. While this limiting issue is being addressed in surface EMG decomposition^50^, in US-based decomposition the lower frequency components of the tissue displacement velocities and the non-linear summation of mechanical responses (twitches)^58,59^ deserves additional studies. We would like to stress that the possibility of generalizing our results is limited by technical issues not associated with the method we propose here. Results similar to those reported here would be expected for different muscles and contraction levels, should we be able to discriminate MUs in HDsEMGs and in US images in these conditions.

The work presented here provides methods to identify and assess the electrical and mechanical MU characteristics, and the associations between spatial location. The identification of MU spatial representation within the muscle allows to assign the decomposed MUAPs to a specific muscle/muscle region, which is relevant to understand the relationship between neural and physical MU properties and can be used for the refinement of musculoskeletal models, as it may provide experimental basis for the definition of the model parameters. While the electrical characteristics and behaviours of MUs have been well studied over previous decades, their physical location and mechanical response to excitation have only just begun to be explored in vivo. This is an important step forward if we are to address acknowledged gaps in understanding of how whole muscles translate the neural commands activating the contractile cells into the contractions that move the skeleton^60^. Information gleaned from multi-modal approaches, such as MU location and territory, is required for us to better investigate both fundamental aspects of MU and muscle function (e.g. to inform enhancement of finite element models of muscle^61^) and changes that result from ageing (e.g. MU loss and emodeling, muscle weakness^62^) or neurodegenerative diseases (e.g. Motor Neurone Diseases^63,64^). Here analysis has focused on the location of the centroids of tissue displacement, however the method provides possibility to establish methods of quantifying the area or spatial contours in the tissue velocity profiles and their association with MU territory. Future work is also required to investigate how factors such as the number of recruited Mus (activation level) and MU firing rate, alongside physical changes in muscle length and tissue properties (e.g. fibrosis, adipose tissue infiltration), influence the physical muscle tissue MU representation. The potential for multi-modal approaches, such as detailed here, to contribute to such investigations is however growing and offer opportunity for such studies to be completed.

## Data Availability

The datasets used and/or analysed during the current study is available from the corresponding author M.C. on reasonable request. The raw data are not publicly available because of the large file sizes.
